# Taxonomic study of the genus *Ischnothyreus* (Araneae, Oonopidae) from Xishuangbanna Rainforest, southwestern China

**DOI:** 10.3897/zookeys.1034.63388

**Published:** 2021-04-26

**Authors:** Yanfeng Tong, Xiaochen Sun, Shuqiang Li, Dongju Bian

**Affiliations:** 1 Life Science College, Shenyang Normal University, Shenyang 110034, China Shenyang Normal University Shenyang China; 2 Institute of Zoology, Chinese Academy of Sciences, Beijing 100101, China Institute of Zoology, Chinese Academy of sciences Beijing China; 3 CAS Key Laboratory of Forest Ecology and Management, Institute of Applied Ecology, Shenyang 110016, China Institute of Applied Ecology, Chinese Academy of Sciences Shenyang China

**Keywords:** Goblin spider, morphology, new species, taxonomy

## Abstract

Eight species of the genus *Ischnothyreus* Simon, 1893 from Xishuangbanna, Yunnan, China are recognized, including six new species: *I. cristiformis* Tong & Li, **sp. nov.** (♂♀), *I. mangun* Tong & Li, **sp. nov.** (♂♀), *I. mengyang* Tong & Li, **sp. nov.** (♂♀), *I. peltifer* (Simon, 1892), *I. qidaoban* Tong & Li, **sp. nov.** (♂♀), *I. qiuxing* Tong & Li, 2020 (♂♀), *I. sijiae* Tong & Li, **sp. nov.** (♀) and *I. xiaolongha* Tong & Li, **sp. nov.** (♂♀). The male of *I. qiuxing* Tong & Li, 2020 is described for the first time. Photos of the habitus and copulatory organs are provided.

## Introduction

Oonopidae Simon, 1890 is a diverse spider family with 1872 extant described species in 114 genera. They have a nearly worldwide distribution but are most abundant in the tropics and subtropics (WSC 2021).

Xishuangbanna is a key biogeographic area and a biodiversity hotspot in China (Myers 1988). Spider diversity in this area is high, with 782 species spanning 305 genera in 46 families (Li 2020). The Oonopidae from this region have been poorly studied, with only 2 genera and 10 species recorded so far (Tong and Li 2015a, 2015b; Sun et al. 2019).

*Ischnothyreus* Simon, 1893 is one of the most speciose genera of the family, with 114 extant species mainly distributed in the Old World (WSC 2021). Up to now, 15 species have been recorded in China (Tong and Li 2008, 2012, 2014; Tong et al. 2018; Liu et al. 2019). In this paper eight *Ischnothyreus* species collected from Xishuangbanna are reported. This work presents the first records and descriptions of species in the genus *Ischnothyreus* from this region.

## Materials and methods

The specimens were examined in 95% ethanol using a Leica M205C stereomicroscope. Details were studied with an Olympus BX51 compound microscope. Photos were taken with a Canon EOS 750D zoom digital camera (18 megapixels) mounted on an Olympus BX51 compound microscope. Vulvae were cleared in lactic acid. Scanning electron microscope images (SEM) were taken under high vacuum with a Hitachi TM3030 after critical point drying and gold-palladium coating. All measurements were taken using an Olympus BX51 compound microscope and are given in millimeters in the text. The specimens are preserved in Shenyang Normal University (SYNU) in Shenyang, China.

The following abbreviations are used in the text and figures: a = apodemes; ALE = anterior lateral eyes; ass = anchor-shaped structure; bsa = bell-shaped atrium; ccp = cockscomb-shaped process; cd = central oval depression; csa = circular atrium; css = central strongly sclerotized structure; fsp = finger-shaped process; hsm = hook-shaped membrane; hsp = helmet-shaped process; llm = lamella-like membrane; lpp = leaf-shaped prolateral projection; lsp = large, sclerotized process; mlp = mushroom-like projection; nsa = nipple-shaped atrium; nsm = needle-shaped membrane; oa = opening of the atrium; PME = posterior median eyes; PLE = posterior lateral eyes; rl = retrolateral lobe; rol = round lobe; rsa = rectangular-shaped atrium; stp = strong, tooth-like projection; tlp = tuber-like projection; tsm = thread-shaped membrane; tsp = tongue-shaped process; vpr = ventral protuberance; wt = winding tube.

## Taxonomy

**Family Oonopidae Simon, 1890**

**Genus *Ischnothyreus* Simon, 1893**

### Ischnothyreus cristiformis

Taxon classificationAnimaliaAraneaeOonopidae

Tong & Li sp. nov.

FE012097-094E-5941-AAFD-FD48C31894FC

http://zoobank.org/F27DFEC6-EED4-4695-A551-1004663BED38

**Figure 1.**
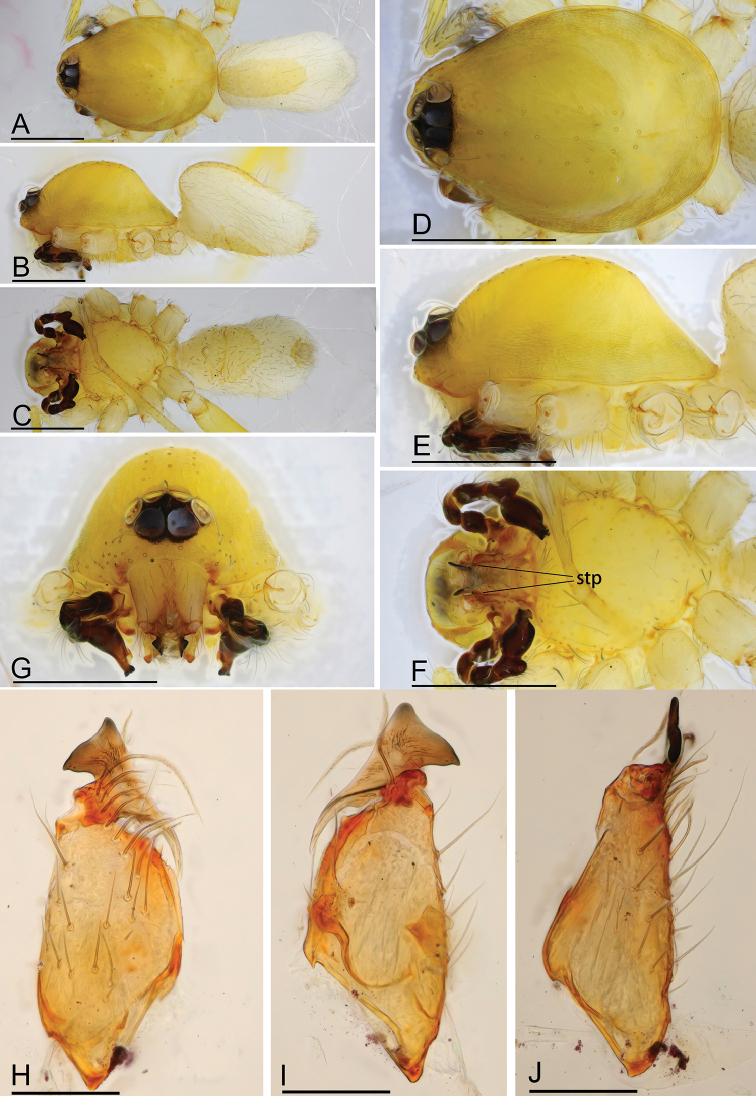
*Ischnothyreus cristiformis* sp. nov., male holotype **A–C** habitus, dorsal, lateral and ventral views **D–G** prosoma, dorsal, lateral, ventral and anterior views **H–J** left chelicerae, anterior, posterior and lateral views. Abbreviation: stp = strong, tooth-like projection. Scale bars: 0.4 mm (**A–G**); 0.1 mm (**H–J**).

**Figure 2.**
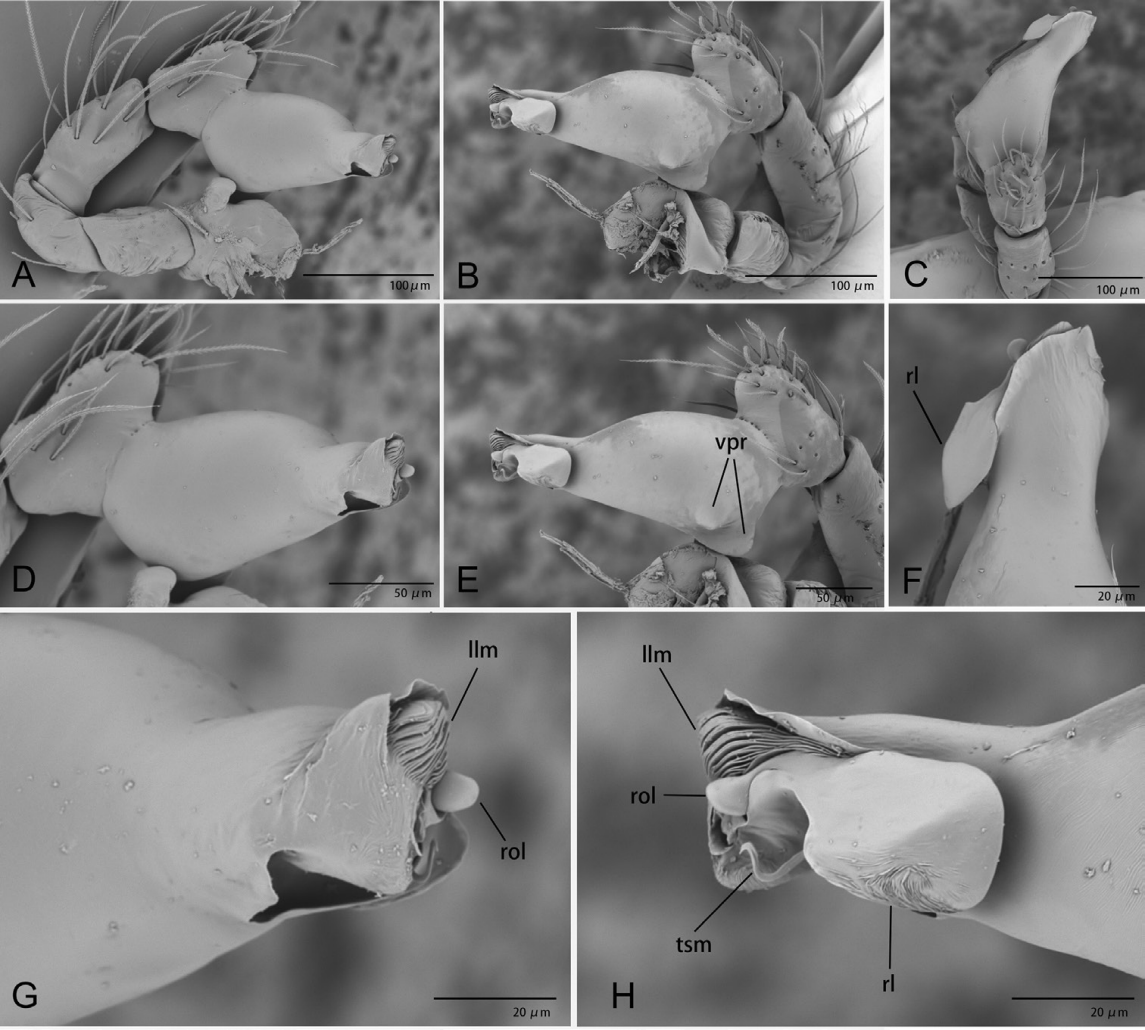
*Ischnothyreus cristiformis* sp. nov., male holotype, left palp, SEM**A–C** prolateral, retrolateral and dorsal views **D, E** palpal bulb, prolateral and retrolateral views **F–H** distal part of palpal bulb, dorsal, prolateral and retrolateral views. Abbreviations: llm = lamella-like membrane; rl = retrolateral lobe; rol = round lobe; tsm = thread-shaped membrane; vpr = ventral protuberance.

**Figure 3.**
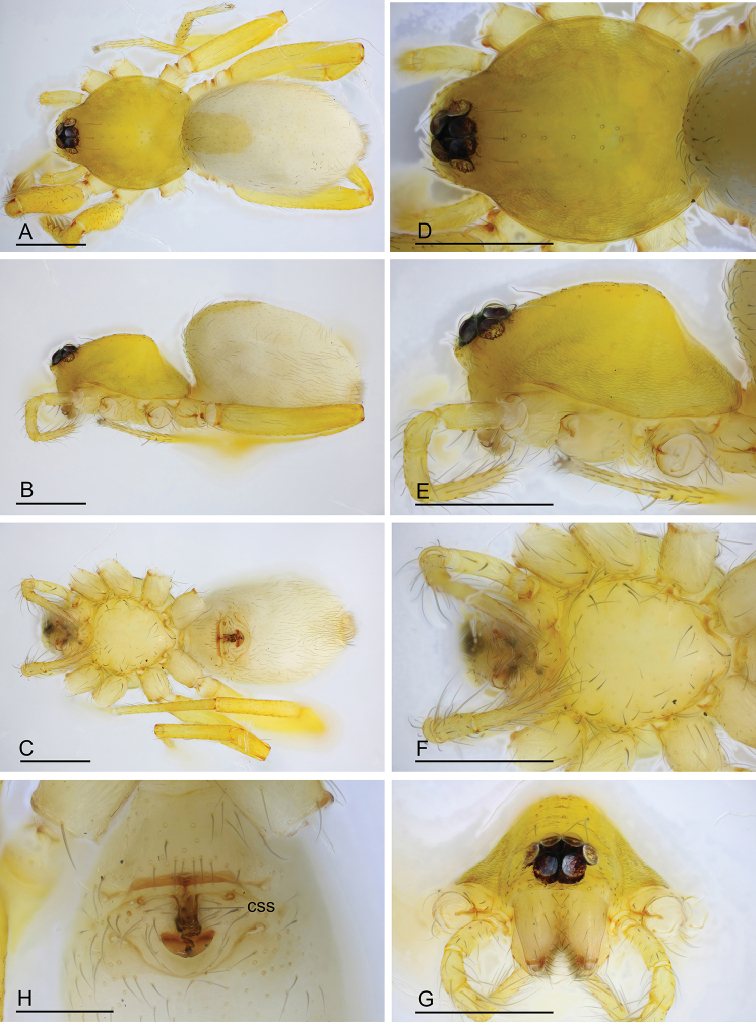
*Ischnothyreus cristiformis* sp. nov., female paratype **A–C** habitus, dorsal, lateral and ventral views **D–G** prosoma, dorsal, lateral, ventral and anterior views **H** epigastric region, ventral view. Abbreviation: css = central strongly sclerotized structure; Scale bars: 0.4 mm (**A–G**); 0.2 mm (**H**).

Figures 1 , 2 , 3 , 20A–C , 22A, B , 23A, B

#### Type material.

***Holotype*** ♂: China, Yunnan, Mengla County, Xiaolongha, Xishuangbanna Biodiversity Conservation Corridor, montane monsoon forest; 21°24.161'N, 101°36.412'E; 791 m; 16.VI.2013; Q. Zhao and Z. Chen leg. (SYNU-379). ***Paratypes.*** 1♀: same data as for holotype (SYNU-380); 3♂1♀: Xiaolongha, Xishuangbanna Biodiversity Conservation Corridor, Qidaoban, valley forest; 21°24.808'N, 101°37.874'E; 711 m; 18.VI.2013; Q. Zhao and Z. Chen leg. (SYNU-381–384).

#### Diagnosis.

**Figure 4.**
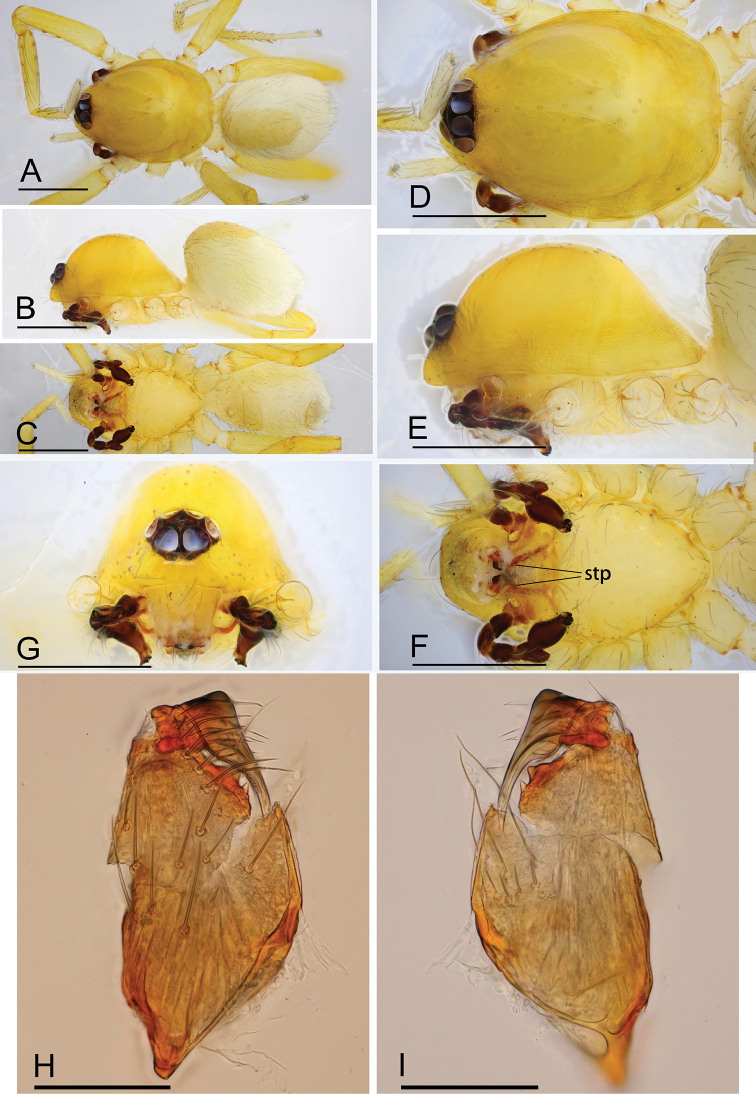
*Ischnothyreus mangun* sp. nov., male holotype **A–C** habitus, dorsal, lateral and ventral views **D–G** prosoma, dorsal, lateral, ventral and anterior views **H, I** left chelicerae, anterior and posterior views. Abbreviation: stp = strong, tooth-like projection. Scale bars: 0.4 mm (**A–G**); 0.1 mm (**H, I**).

**Figure 5.**
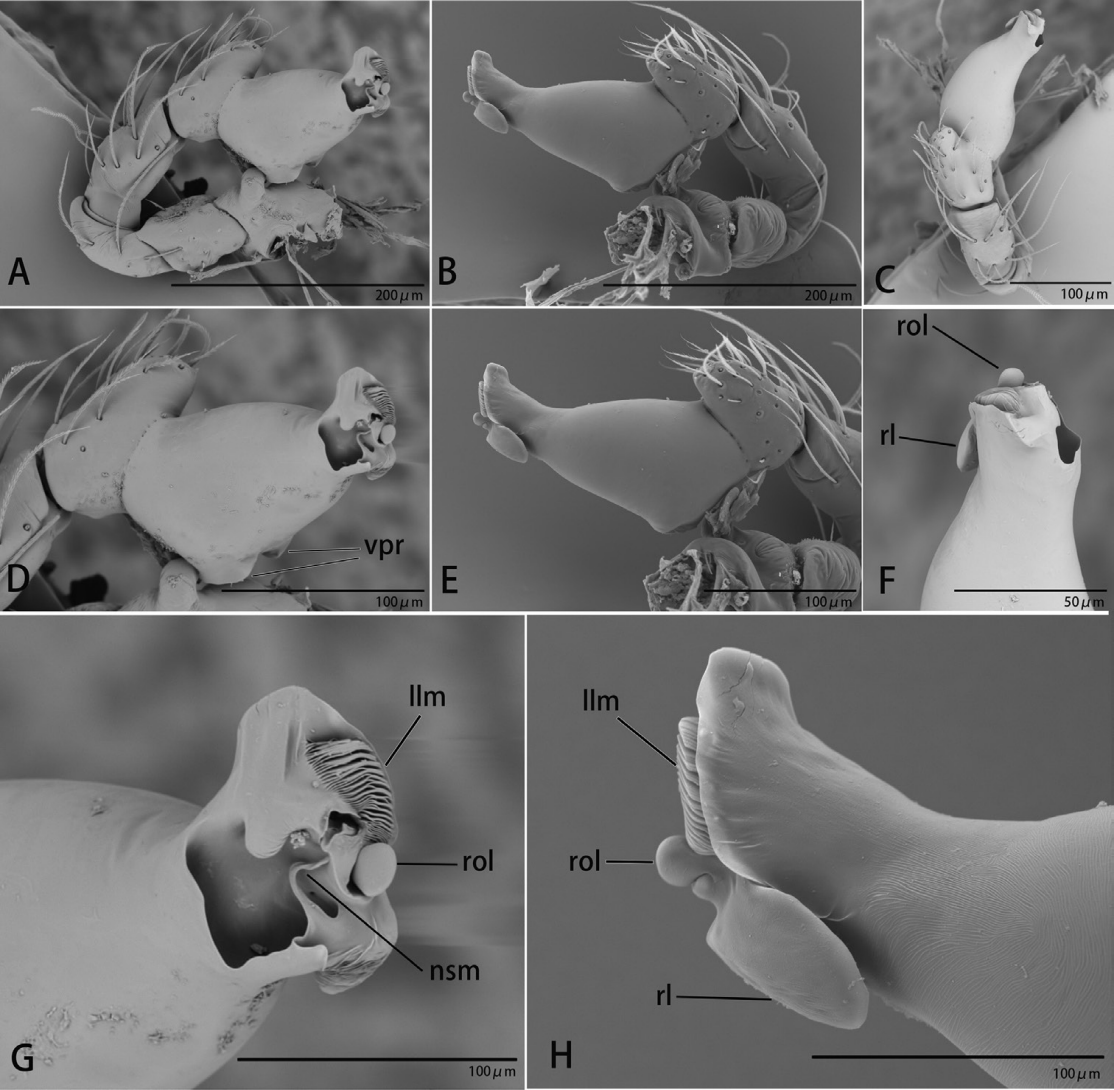
*Ischnothyreus mangun* sp. nov., male holotype, left palp, SEM**A–C** prolateral, retrolateral and dorsal views **D, E** palpal bulb, prolateral and retrolateral views **F–H** distal part of palpal bulb, dorsal, prolateral and retrolateral views. Abbreviations: llm = lamella-like membrane; nsm = needle-shaped membrane; rl = retrolateral lobe; rol = round lobe; vpr = ventral protuberance.

**Figure 6.**
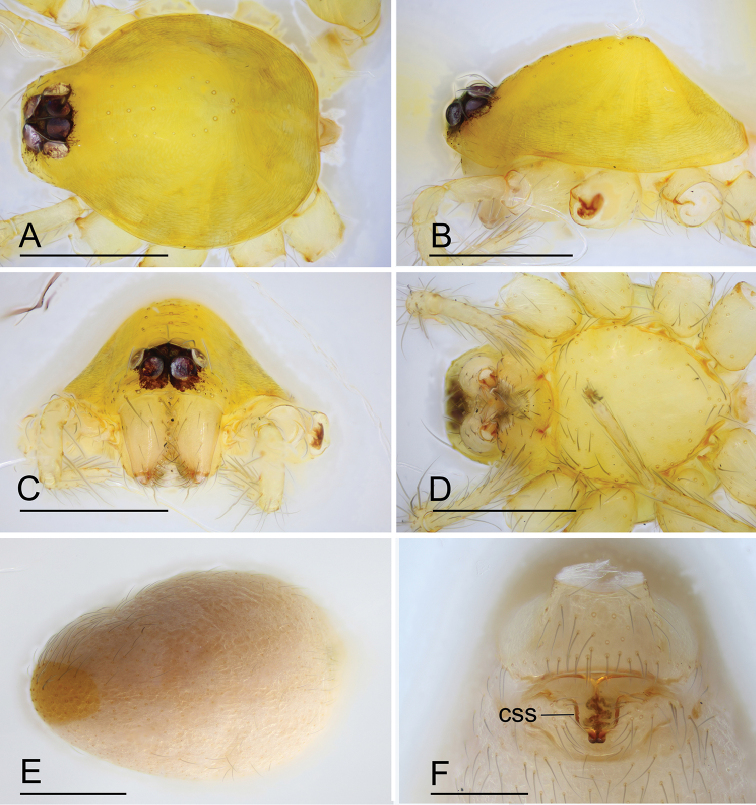
*Ischnothyreus mangun* sp. nov., female paratype **A–D** prosoma, dorsal, lateral, anterior and ventral views **E** abdomen, dorsal view **F** epigastric region, ventral view. Abbreviation: css = central strongly sclerotized structure. Scale bars: 0.4 mm (**A–E**); 0.2 mm (**F**).

The new species is similar to *I. mangun* sp. nov. and *I. xiaolongha* sp. nov. by the lamella-like membrane of the male palp and short dorsal abdominal scutum, but can be distinguished by the large cockscomb-shaped sclerotized process (Fig. 1H–J) of the male cheliceral fang (vs the unmodified cheliceral fang (Fig. 4H, I) in *I. mangun* sp. nov. and the tongue-shaped sclerotized process (Fig. 17H–J) in *I. xiaolongha* sp. nov.), the broad rectangular-shaped retrolateral lobe of the bulb (Fig. 2F) (vs the leaf-shaped lobe (Figs 5F, 18F) in *I. mangun* sp. nov. and *I. xiaolongha* sp. nov.). The female differs from *I. mangun* sp. nov. and *I. xiaolongha* sp. nov. by the large bowl-shaped atrium (Fig. 3H) (vs small rectangular-shaped atrium (Fig. 6F) in *I. mangun* sp. nov. and small bell-shaped atrium (Fig. 19H) in *I. xiaolongha* sp. nov.).

#### Description.

**Male (holotype). *Body***: habitus as in Fig. 1A–C; body length 1.64. ***Carapace***: 0.88 long, 0.65 wide; yellow, with egg-shaped patches behind eyes, surface of elevated portion of pars cephalica smooth, sides finely reticulate, lateral margin straight, smooth (Fig. 1D, E). ***Clypeus***: height about 2/3 of ALE diameter (Fig. 1G). ***Eyes***: see Fig. 1D, G. ***Sternum***: pale orange (Fig. 1F). ***Mouthparts***: chelicerae, endites and labium orange; chelicerae straight, base of fangs with large cockscomb-shaped sclerotized process, fang groove with a few small denticles (Figs 1H–J, 22A, B); anteromedian tip of endites with one strong, tooth-like projection (Fig. 1F). ***Abdomen***: 0.76 long, 0.44 wide; dorsal scutum well sclerotized, pale orange, covering 1/3 of the abdomen width and approximately 2/3 of the abdomen length, fused to epigastric scutum; epigastric and postgastric scute well sclerotized, pale orange, fused, postgastric scutum covering about 2/3 of the abdomen length (Fig. 1A–C). ***Legs***: pale orange, femur I with 2 prolateral spines, tibia I with 4 pairs, metatarsus I with 2 pairs of long ventral spines. Leg II spination similar to leg I except femur with only 1 prolateral spine. Legs III and IV spineless. ***Palp***: trochanter with ventral projection, cymbium brown; bulb with 2 ventral protuberances, one large and another very small, distal end of bulb elongated, with numerous lamella-like membranes, retrolateral lobe broad, rectangular-shaped (Figs 2, 20A–C).

**Female (paratype, SYNU-380).** Same as male except as noted. ***Body***: habitus as in Fig. 3A–C; body length 1.76. **Carapace**: 0.72 long, 0.67 wide. ***Mouthparts***: chelicerae and endites unmodified. ***Abdomen***: 1.05 long, 0.67 wide; dorsal scutum covering 1/3 of the abdomen length, about 1/4 of the abdomen width. ***Epigastric area***: postgastric scutum with strongly sclerotized structure in the middle (Fig. 3H). ***Endogyne***: from the middle of the slightly thickened margin of the postgastric scutum runs a dark, simple winding tube, ending in a large bowl-shaped atrium (Fig. 23A, B).

#### Etymology.

The specific name is a Latin adjective and refers to the large cockscomb-shaped sclerotized process of the cheliceral fang.

#### Distribution.

Known only from the localities of the type series.

### Ischnothyreus mangun

Taxon classificationAnimaliaAraneaeOonopidae

Tong & Li sp. nov.

D5330EE2-985D-5002-8BF3-A227CC9D681F

http://zoobank.org/0A789285-4F33-40FB-9B2A-8C2A5F165345

Figures 4 , 5 , 6 , 20D–F , 22C, D , 23C, D

#### Type material.

***Holotype*** ♂: China, Yunnan, Menghai County, Mangun Stockaded Village, Xishuangbanna Natural Reserve, secondary forest; 22°02’12’’N, 100°23’28’’E; 1179 m; 20.III.2016; S. Li leg. (SYNU-385). ***Paratype.*** 1♀: same data as for holotype (SYNU-386).

#### Diagnosis.

The new species is similar to *I. cristiformis* sp. nov. and *I. xiaolongha* sp. nov. in the lamella-like membrane of male palp and the short dorsal scutum of the abdomen, but can be distinguished by the unmodified male cheliceral fang (Fig. 4H, I) (vs the large cockscomb-shaped sclerotized process (Fig. 1H–J) in *I. cristiformis* sp. nov. and the tongue-shaped sclerotized process (Fig. 17H–J) in *I. xiaolongha* sp. nov.), the narrow leaf-shaped retrolateral lobe of the male palp (Fig. 5F) (vs broad rectangular-shaped (Fig. 2F) in *I. cristiformis* sp. nov. and broad leaf-shaped (Fig. 18F) in *I. xiaolongha* sp. nov.). The female differs from *I. cristiformis* and *I. xiaolongha* by the small rectangular-shaped atrium (Fig. 6F) (vs the large bowl-shaped atrium (Fig. 3H) in *I. cristiformis* sp. nov. and small bell-shaped atrium (Fig. 19H) in *I. xiaolongha* sp. nov.).

#### Description.

**Male (holotype). *Body***: habitus as in Fig. 4A–C; body length 1.52. ***Carapace***: 0.81 long, 0.61 wide; yellow, with egg-shaped patches behind eyes, surface of elevated portion of pars cephalica smooth, sides finely reticulate, lateral margin straight, smooth (Fig. 4D, E). ***Clypeus***: height about 1.3 times ALE diameter (Fig. 4G). ***Eyes***: see Fig. 4D, G. ***Sternum***: pale orange (Fig. 4F). ***Mouthparts***: chelicerae, endites and labium orange; chelicerae straight, base of fangs unmodified, fang groove with a few small denticles (Figs 4H, I, 22C, D); anteromedian tip of endites with one strong, tooth-like projection (Fig. 4F). ***Abdomen***: 0.65 long, 0.49 wide; dorsal scutum well sclerotized, pale orange, covering 1/3 of the abdomen width and approximately 1/2 of the abdomen length, not fused to epigastric scutum; epigastric and postgastric scutum well sclerotized, pale orange, fused, postgastric scutum covering about 1/2 of the abdomen length (Fig. 4A–C). ***Legs***: pale orange, femur I with 2 prolateral spines, tibia I with 4 pairs, metatarsus I with 2 pairs of long ventral spines. Legs II lost. Legs III and IV spineless. ***Palp***: trochanter with ventral projection, cymbium brown; bulb with 2 ventral protuberances, one large and another very small, distal end of bulb stout, with numerous lamella-like membranes, retrolateral lobe narrow leaf-shaped (Figs 5, 20D–F).

**Female (paratype, SYNU-386).** Same as male except as noted. ***Body***: body length 1.93. ***Carapace***: 0.80 long, 0.64 wide. ***Mouthparts***: chelicerae and endites unmodified. ***Abdomen***: 1.20 long, 0.81 wide; dorsal scutum very small. ***Epigastric area***: the postgastric scutum with a strongly sclerotized structure in the middle (Fig. 6F). ***Endogyne***: from the middle of the slightly thickened margin of the postgastric scutum runs a dark, simple, winding tube posteriorly, ending in a small rectangular shaped atrium (Fig. 23C, D).

#### Etymology.

The specific name is a noun in apposition taken from the type locality.

#### Distribution.

Known only from the type locality.

### Ischnothyreus mengyang

Taxon classificationAnimaliaAraneaeOonopidae

Tong & Li sp. nov.

86134189-3F13-5E22-8B23-D9194E516BD3

http://zoobank.org/544C1DAA-EB94-4707-B4A5-688047F8ABD9

**Figure 7.**
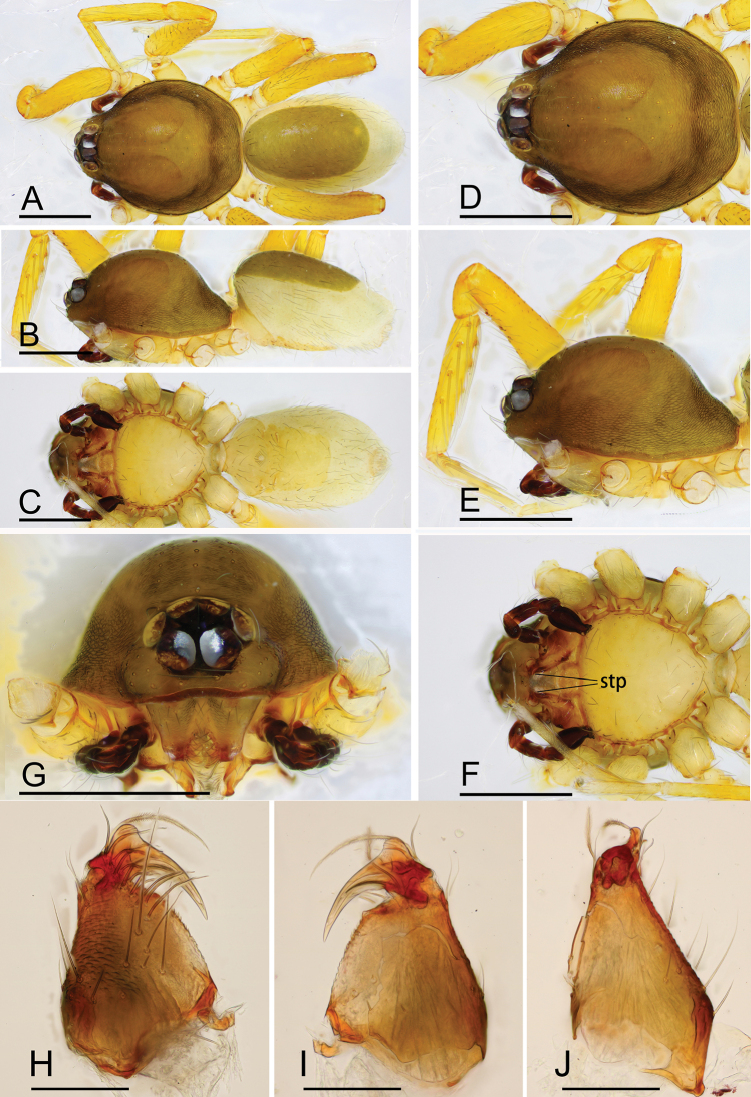
*Ischnothyreus mengyang* sp. nov., male holotype **A–C** habitus, dorsal, lateral and ventral views **D–G** prosoma, dorsal, lateral, ventral and anterior views **H–J** left chelicerae, anterior, posterior and lateral views. Abbreviation: stp = strong, tooth-like projection. Scale bars: 0.4 mm (**A–G**); 0.1 mm (**H–J**).

**Figure 8.**
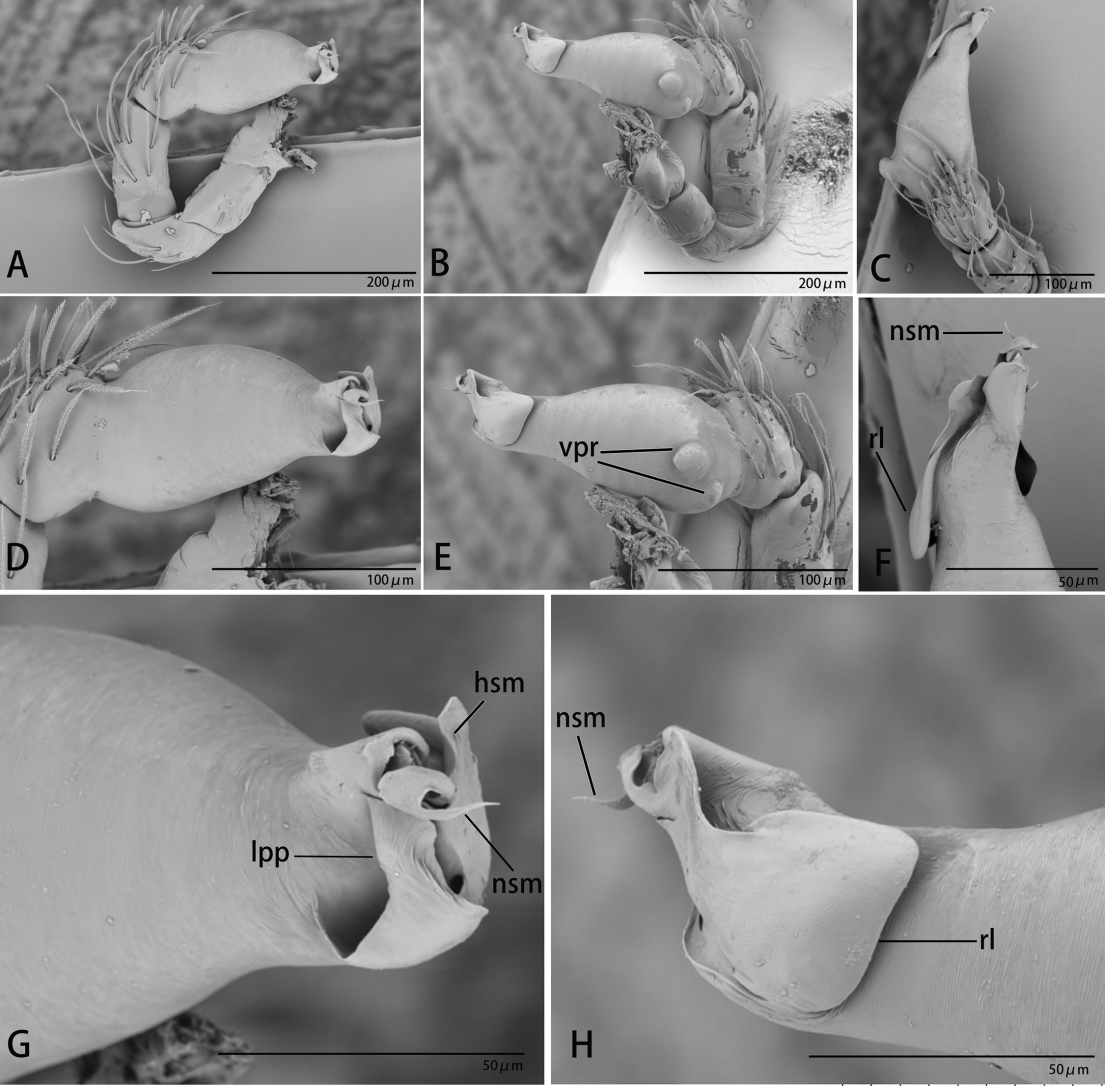
*Ischnothyreus mengyang* sp. nov., male holotype, left palp, SEM**A–C** prolateral, retrolateral and dorsal views **D, E** palpal bulb, prolateral and retrolateral views **F–H** distal part of palpal bulb, dorsal, prolateral and retrolateral views. Abbreviations: hsm = hook-shaped membrane; lpp = leaf-shaped prolateral projection; nsm = needle-shaped membrane; rl = retrolateral lobe; vpr = ventral protrusion.

**Figure 9.**
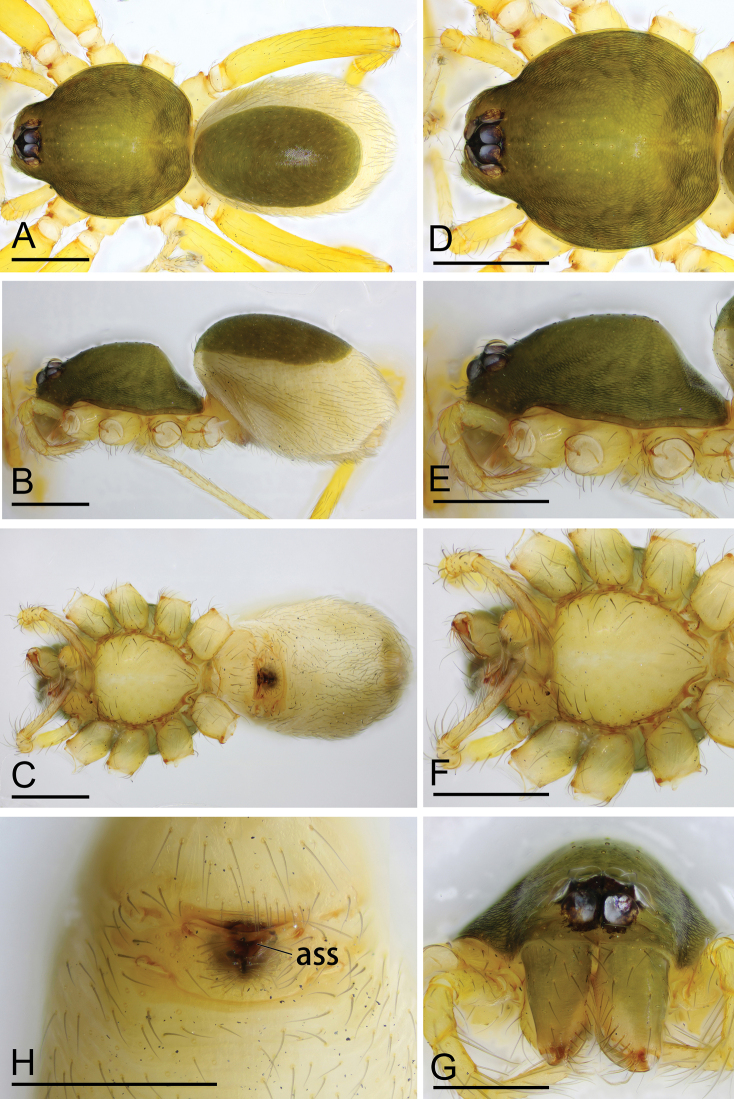
*Ischnothyreus mengyang* sp. nov., female paratype **A–C** habitus, dorsal, lateral and ventral views **D–G** prosoma, dorsal, lateral, ventral and anterior views **H** epigastric region, ventral view. Abbreviation: ass = anchor-shaped structure. Scale bars: 0.4 mm (**A–H**).

Figures 7 , 8 , 9 , 20G–I , 22E, F , 23E, F

#### Type material.

***Holotype*** ♂: China, Yunnan, Jinghong City, Mengyang Town, Xishuangbanna Natural Reserve, monsoon forest; 21°24.161'N, 101°36.412'E; 791 m; 16.VI.2013; Q. Zhao and Z. Chen leg. (SYNU-387). ***Paratypes*** 5♂12♀: same data as for holotype (SYNU-388–404).

#### Diagnosis.

The new species is similar to *I. taunggyi* Tong & Li, 2020 in the male palp and the large, sclerotized process of male cheliceral fang, but can be distinguished by the broad retrolateral lobe of male palp (Fig. 8F) (vs small, ear-shaped retrolateral lobe in *I. taunggyi*; Tong et al. 2020: fig. 9F) and by the long abdominal dorsal scutum of the female (5/6 of the abdomen length (Fig. 9A) vs less than 1/2 of the abdomen length in *I. taunggyi*; Tong et al. 2020: fig. 10A).

#### Description.

**Male (holotype). *Body***: habitus as in Fig. 7A–C; body length 1.86. ***Carapace***: 0.95 long, 0.78 wide; pale brown, with egg-shaped patches behind eyes, surface of elevated portion of pars cephalica smooth, sides finely reticulate, lateral margin straight, smooth (Fig. 7D, E). ***Clypeus***: height about 0.74 times ALE diameter (Fig. 7G). ***Eyes***: see Fig. 7D, G. ***Sternum***: pale orange (Fig. 7F). ***Mouthparts***: chelicerae, endites and labium brown; chelicerae straight, base of fangs with large, sclerotized process, fang groove with a few small denticles (Figs 7H–J, 22E, F); anteromedian tip of endites with one strong, tooth-like projection (Fig. 7F). ***Abdomen***: 0.92 long, 0.55 wide; dorsal scutum well sclerotized, pale orange, covering approximately 4/5 of the abdomen length and 3/4 of the abdomen width, not fused to epigastric scutum; epigastric and postgastric scutum well sclerotized, pale orange, fused, postgastric scutum covering about 3/4 of the abdomen length (Fig. 7A–C). ***Legs***: pale orange, femur I with 2 prolateral spines, tibia I with 4 pairs, metatarsus I with 2 pairs of long ventral spines. Leg II spination similar to leg I except femur with only 1 prolateral spine. Legs III and IV spineless. ***Palp***: trochanter with ventral projection, cymbium brown; bulb with 2 ventral protuberances, one large and another very small, distal end of bulb elongated, with one narrow leaf-shaped prolateral projection and a distal hook-shaped membrane, retrolateral lobe broad (Figs 8, 20G–I).

**Female (paratype, SYNU-393).** Same as male except as noted. ***Body***: habitus as in Fig. 9A–C; body length 1.96. ***Carapace***: 0.94 long, 0.82 wide. ***Mouthparts***: chelicerae and endites unmodified. ***Abdomen***: 1.05 long, 0.73 wide; dorsal scutum covering 5/6 of the abdomen length, about 2/3 of the abdomen width. ***Epigastric area***: postgastric scutum with central anchor-shaped structure (Fig. 9H). ***Endogyne***: from the middle of the slightly thickened margin of the postgastric scutum runs a dark, complex winding tube, ending in a very small nipple-shaped atrium (Fig. 23E, F).

#### Etymology.

The specific name is a noun in apposition taken from the type locality.

#### Distribution.

Known only from the type locality.

### Ischnothyreus peltifer

Taxon classificationAnimaliaAraneaeOonopidae

(Simon, 1892)

FDEB7CA9-2C62-5EA6-BCCF-45B99AD71CE3

Ischnaspis peltifer Simon, 1892: 562.
Ischnothyreus peltifer : Platnick et al. 2012: 7, figs 1–99; Brescovit et al. 2019: 8, figs 20–25.

#### Material examined.

5♂7♀: China, Yunnan, Xishuangbanna Tropical Garden; 21°54.999'N, 101°16.237'E; 561 m; 24.IV.2019; Y. Tong and J. Liu leg. (SYNU-414–425).

#### Diagnosis.

See Platnick et al. (2012).

#### Distribution.

Tropical Asia. Introduced to North, Central, South America, Britain, Gaboon, Seychelles, Madagascar, Hawaii.

### Ischnothyreus qidaoban

Taxon classificationAnimaliaAraneaeOonopidae

Tong & Li sp. nov.

06E0F524-B435-5589-9EF6-D994FB002536

http://zoobank.org/C69A3DF7-810D-46A9-B76A-73DF6E02D327

**Figure 10.**
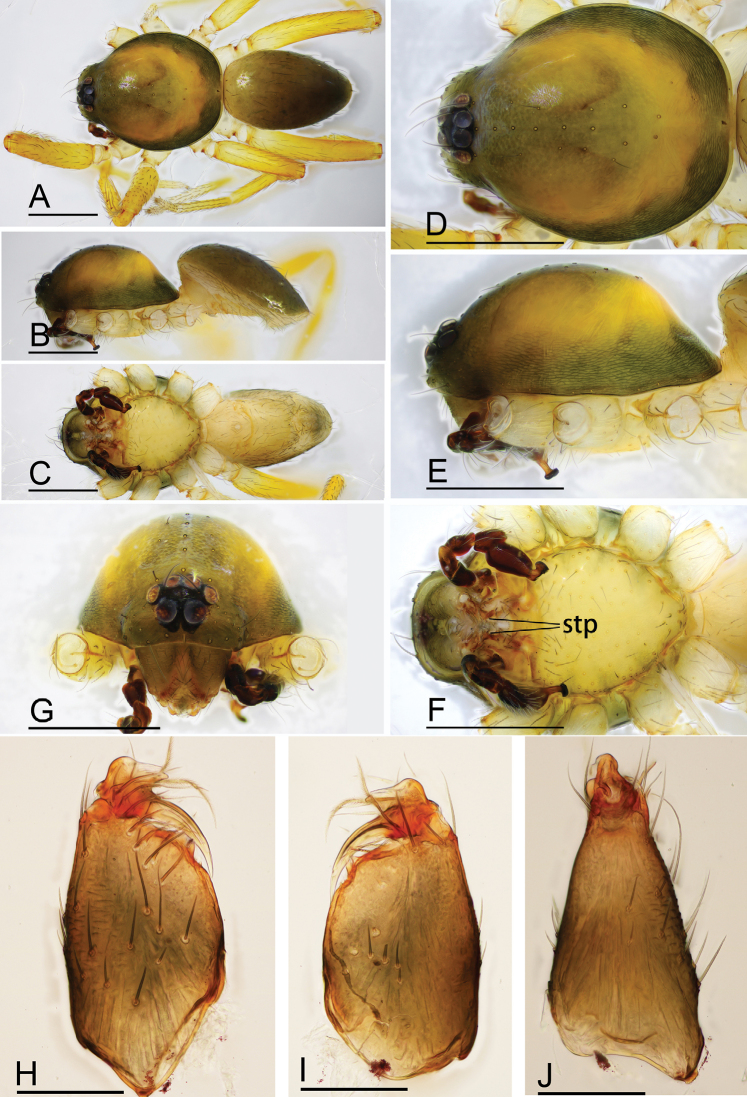
*Ischnothyreus qidaoban* sp. nov., male holotype **A–C** habitus, dorsal, lateral and ventral views **D–G** prosoma, dorsal, lateral, ventral and anterior views **H–J** left chelicerae, anterior, posterior and lateral views. Abbreviation: stp = strong, tooth-like projection. Scale bars: 0.4 mm (**A–G**); 0.1 mm (**H–J**).

**Figure 11.**
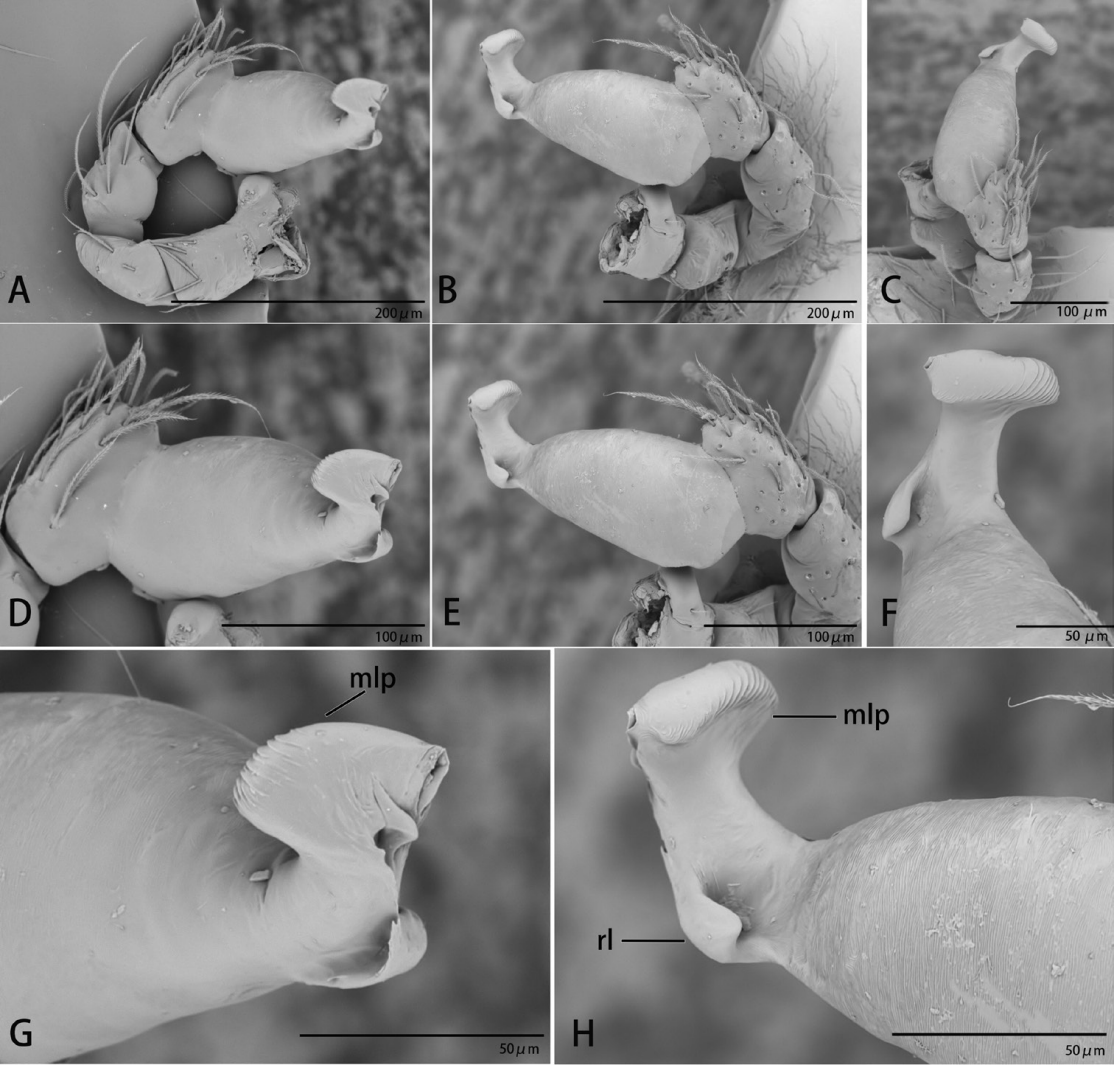
*Ischnothyreus qidaoban* sp. nov., male holotype, left palp, SEM**A–C** prolateral, retrolateral and dorsal views **D, E** palpal bulb, prolateral and retrolateral views **F–H** distal part of palpal bulb, dorsal, prolateral and retrolateral views. Abbreviations: mlp = mushroom-like projection; rl = retrolateral lobe.

**Figure 12.**
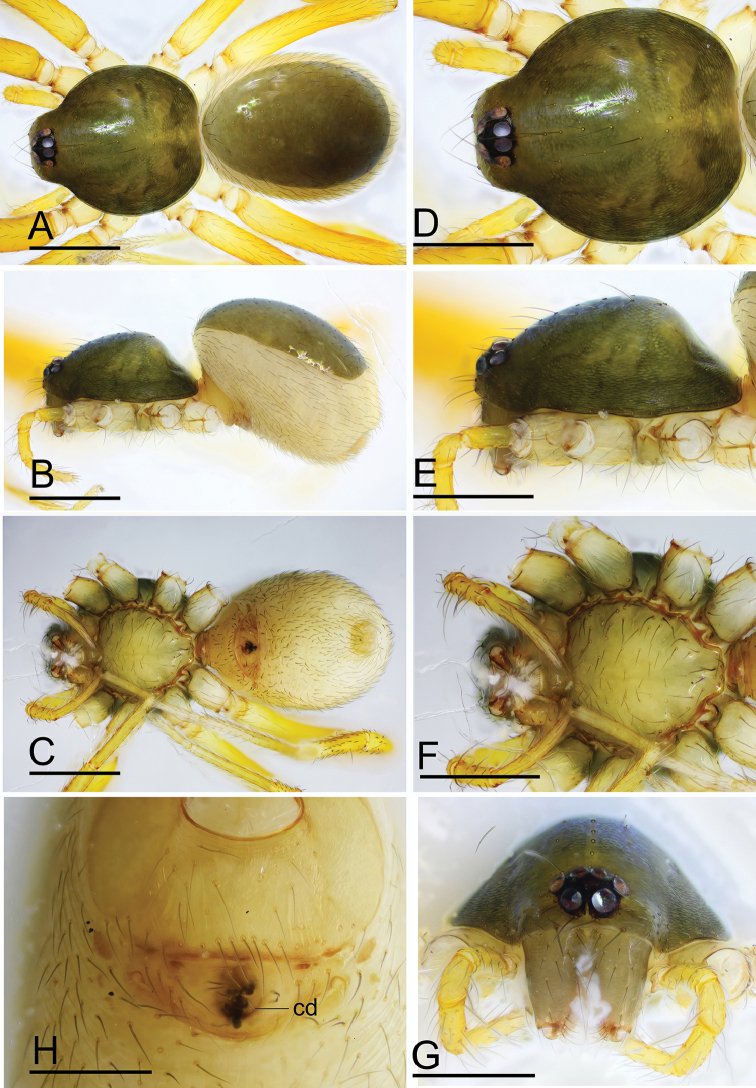
*Ischnothyreus qidaoban* sp. nov., female paratype **A–C** habitus, dorsal, lateral and ventral views **D–G** prosoma, dorsal, lateral, ventral and anterior views **H** epigastric region, ventral view. Abbreviation: cd = central oval depression. Scale bars: 0.4 mm (**A–G**); 0.2 mm (**H**).

Figures 10 , 11 , 12 , 20J–L , 22G, H , 23G, H

#### Type material.

***Holotype*** ♂: China, Yunnan, Mengla City, Menglun Town, Xishuangbanna Natural Reserve, 55 Km, monsoon forest; 21°57.531'N, 101°11.961'E; 751 m; 13.VI.2013; Q. Zhao and Z. Chen leg. (SYNU-405). ***Paratypes.*** 1♂5♀: same data as for holotype (SYNU-406–411); 1♂1♀: Xiaolongha, Xishuangbanna Biodiversity Conservation Corridor, Qidaoban, valley forest; 21°24.808'N, 101°37.874'E; 711 m; 18.VI.2013; Q. Zhao and Z. Chen leg. (SYNU-412–413).

#### Diagnosis.

The new species is similar to *I. auritus* Tong & Li, 2012 in the long dorsal scutum of the abdomen and the helmet-shaped sclerotized process of the male cheliceral fang, but can be distinguished by the broad mushroom-like projection (Fig. 20J–L) of the palp (vs the ear-shaped apophysis in *I. auritus*; Tong 2013: fig. 43A, B) and the central oval shaped depression (Figs 12H, 23G) of epigastric area (vs without external features in *I. auritus*; Tong 2013: fig. 43C).

#### Description.

**Male (holotype). *Body***: habitus as in Fig. 10A–C; body length 1.58. ***Carapace***: 0.84 long, 0.69 wide; dark brown, with egg-shaped patches behind eyes, surface of elevated portion of pars cephalica finely reticulate, sides strongly reticulate, lateral margin straight, smooth (Fig. 10D, E). ***Clypeus***: height about equal to ALE diameter (Fig. 10G). ***Eyes***: (Fig. 10D, G). ***Sternum***: pale orange (Fig. 10F). ***Mouthparts***: chelicerae, endites and labium brown; chelicerae straight, base of fangs with helmet-shaped sclerotized process, fang groove with a few small denticles (Figs 10H–J, 22G, H); anteromedian tip of endites with one strong, tooth-like projection (Fig. 10F). ***Abdomen***: 0.74 long, 0.46 wide; dorsal scutum well sclerotized, pale orange, covering whole abdomen width and abdomen length, fused to epigastric scutum; epigastric and postgastric scutum well sclerotized, yellow, fused, postgastric scutum covering about 5/6 of the abdomen length (Fig. 10A–C). ***Legs***: yellow, femur I with 2 prolateral spines, tibia I with 4 pairs, metatarsus I with 2 pairs of long ventral spines. Leg II spination similar to leg I except femur with only 1 prolateral spine. Legs III and IV spineless. ***Palp***: trochanter with ventral projection, cymbium brown; bulb with one small ventral protuberance, distal end of bulb elongated, with one broad mushroom-like projection, retrolateral lobe narrow (Figs 11, 20J–L).

**Female (paratype, SYNU-407).** Same as male except as noted. ***Body***: habitus as in Fig. 12A–C; body length 1.64. ***Carapace***: 0.77 long, 0.67 wide. ***Mouthparts***: chelicerae and endites unmodified. ***Abdomen***: 0.88 long, 0.65 wide; dorsal scutum covering 5/6 of the abdomen length and about 5/6 of the abdomen width. ***Epigastric area***: postgastric scutum with central oval-shaped depression (Fig. 12C, H). ***Endogyne***: from the middle of the slightly thickened margin of the postgastric scutum runs a dark, simple winding tube posteriorly (Fig. 23G, H).

#### Etymology.

The specific name is a noun in apposition taken from the type locality.

#### Distribution.

Known only from the localities of the type series.

### Ischnothyreus qiuxing

Taxon classificationAnimaliaAraneaeOonopidae

Tong & Li, 2020

B02F992B-F5EE-55D5-BB42-C58EDBDF4C71

**Figure 13.**
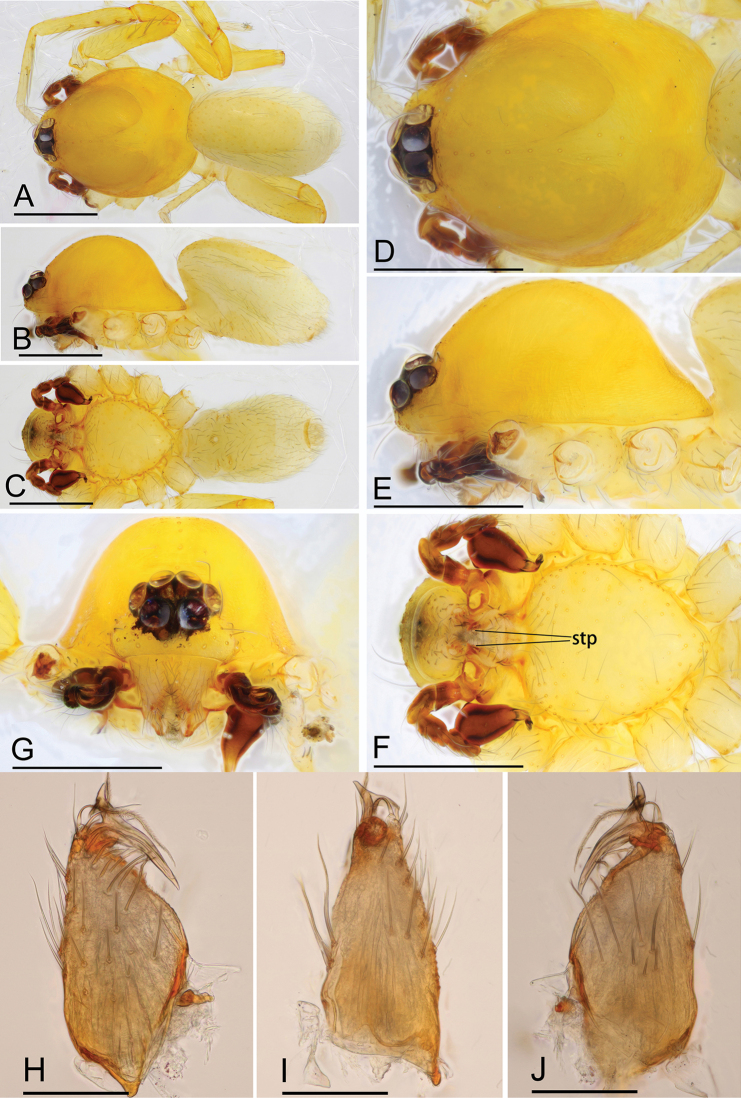
*Ischnothyreus qiuxing*, male (SYNU-372) **A–C** habitus, dorsal, lateral and ventral views **D–G** prosoma, dorsal, lateral, ventral and anterior views **H–J** left chelicerae, anterior, lateral and posterior views. Abbreviation: stp = strong, tooth-like projection. Scale bars: 0.4 mm (**A–G**); 0.1 mm (**H–J**).

**Figure 14.**
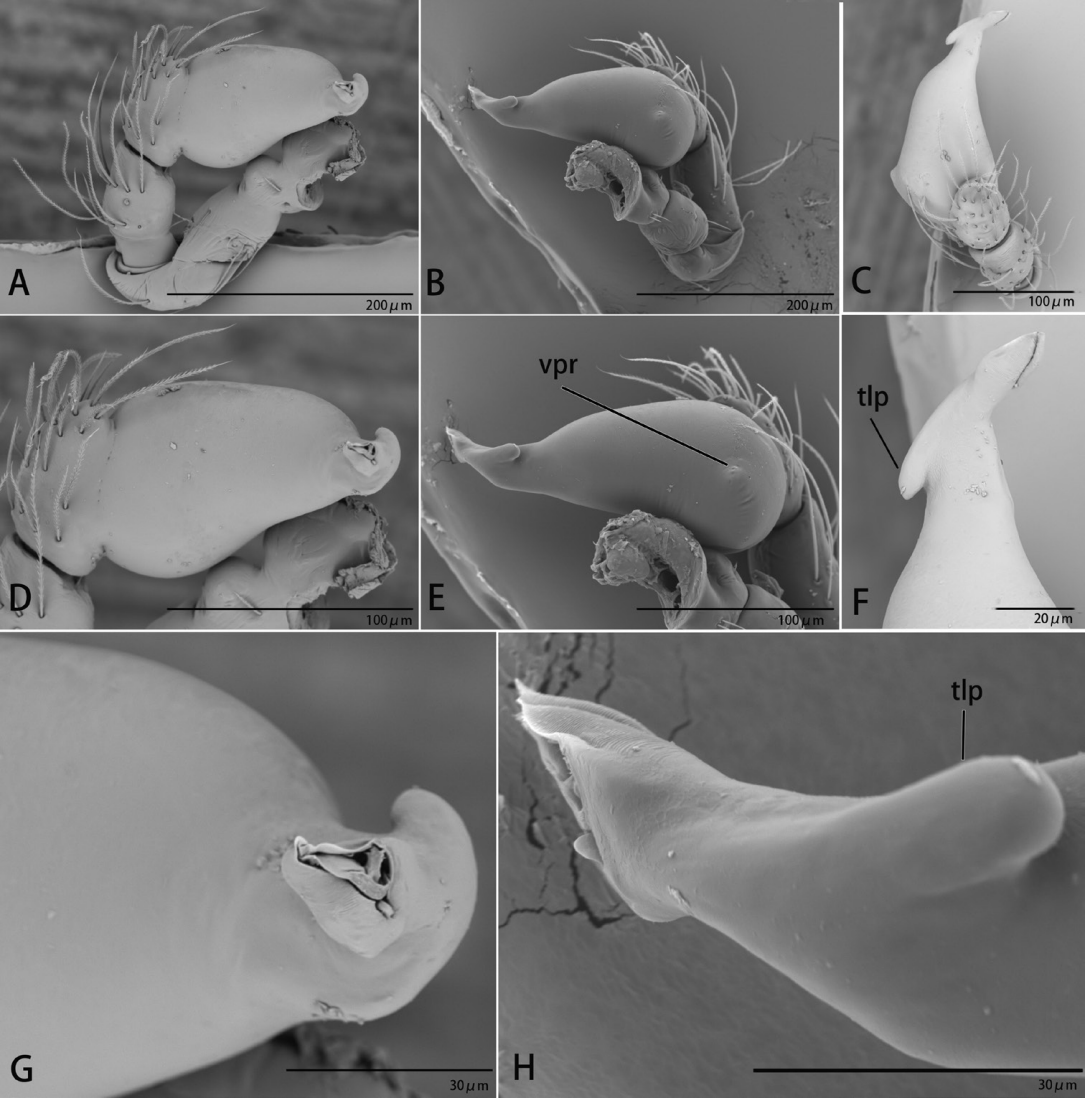
*Ischnothyreus qiuxing*, male (SYNU-372), left palp, SEM**A–C** prolateral, retrolateral and dorsal views **D, E** palpal bulb, prolateral and retrolateral views **F–H** distal part of palpal bulb, dorsal, prolateral and retrolateral views. Abbreviations: tlp = tuber-like projection; vpr = ventral protrusion.

**Figure 15.**
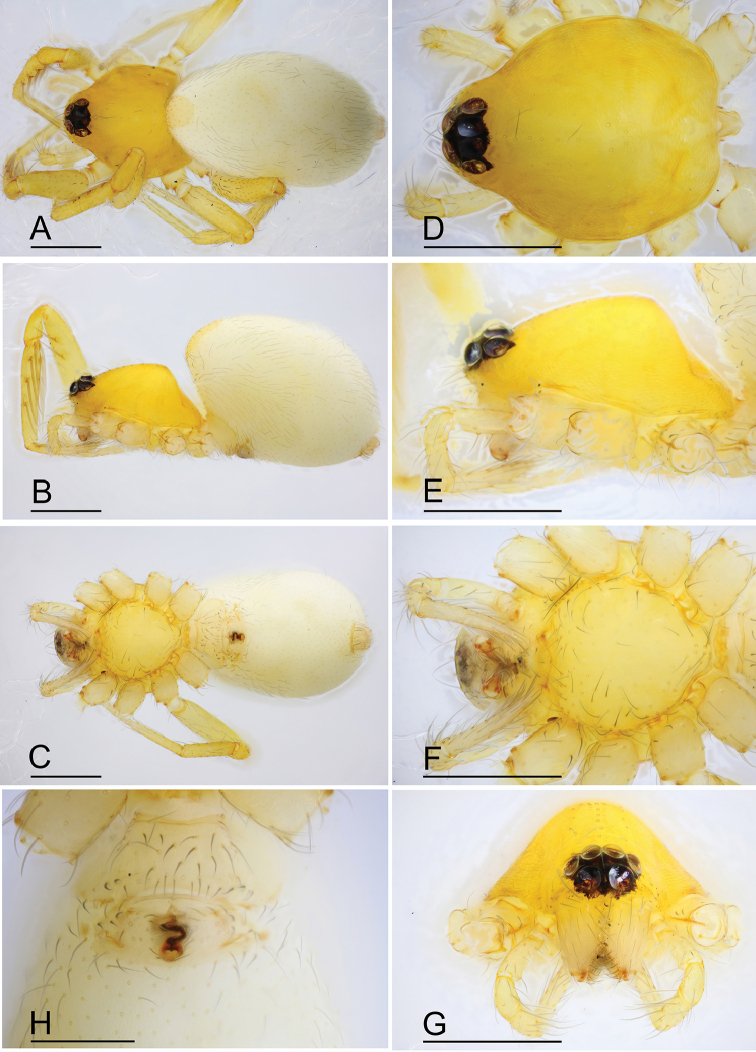
*Ischnothyreus qiuxing*, female (SYNU-378) **A–C** habitus, dorsal, lateral and ventral views **D–G** prosoma, dorsal, lateral, ventral and anterior views **H** epigastric region, ventral view. Scale bars: 0.4 mm (**A–G**); 0.2 mm (**H**).

Figures 13 , 14 , 15 , 20M–O , 21F, G , 23I, J

Ischnothyreus qiuxing Tong & Li, 2020: 12, figs 7A–H, 16E, F.

#### Material examined.

4♂3♀: China, Yunnan, Menghai City, Mangun Stockaded Village, Xishuangbanna Natural Reserve, secondary forest; 22°02’12’’N, 100°23’28’’E; 1179 m; 20.III.2016; S. Li leg. (SYNU-372–378).

#### Diagnosis.

This species is similar to *I. balu* Kranz-Baltensperger, 2011 in the circular atrium in the female, but can be distinguished by the finger-shaped sclerotized process of the male cheliceral fang (Fig. 13H–J) (lacking in *I. balu*; Kranz-Baltensperger 2011: fig. 1F–G), the size of the atrium (nearly 1/5 the length of postgastric scutum (Fig. 23I)) (vs more than 1/3 the length of postgastric scutum; Kranz-Baltensperger 2011: fig. 2F, H) and the greater sinuosity of the winding tube (Fig. 23J) (vs the short, simple winding tube in *I. balu*; Kranz-Baltensperger 2011: fig. 2G).

#### Redescription.

**Male (SYNU-372). *Body***: habitus as in Fig. 13A–C; body length 1.90. ***Carapace***: 1.03 long, 0.81 wide; yellow, with egg-shaped patches behind eyes, surface of elevated portion of pars cephalica smooth, sides finely reticulate, lateral margin straight, smooth (Fig. 13D, E). ***Clypeus***: height about equal to ALE diameter (Fig. 13G). ***Eyes***: see Fig. 13D, G. ***Sternum***: pale orange (Fig. 13F). ***Mouthparts***: chelicerae, endites and labium orange; chelicerae straight, base of fangs with finger-shaped sclerotized process, fang groove with a few small denticles (Figs 13H–J, 21F, G); anteromedian tip of endites with one strong, tooth-like projection (Fig. 13F). ***Abdomen***: 0.95 long, 0.54 wide; dorsal scutum well sclerotized, pale orange, covering approximately 2/3 of the abdomen width and 3/4 of the abdomen length, fused to epigastric scutum; epigastric and postgastric scutum well sclerotized, pale orange, fused, postgastric scutum covering about 3/4 of the abdomen length (Fig. 13A–C). ***Legs***: pale orange, femur I with 2 prolateral spines, tibia I with 4 pairs, metatarsus I with 2 pairs of long ventral spines. Leg II spination similar to leg I except femur with only 1 prolateral spine. Legs III and IV spineless. ***Palp***: trochanter with ventral projection, cymbium brown; bulb with one very small ventral protuberance, distal end of bulb elongated, with distal narrow membrane, and a retrolateral tuber-like projection (Figs 14, 20M–O).

**Female (redescription, SYNU-378).** Same as male except as noted. ***Body***: habitus as in Fig. 15A–C; body length 1.82. ***Carapace***: 0.81 long, 0.66 wide. ***Mouthparts***: chelicerae and endites unmodified. ***Abdomen***: 1.24 long, 0.79 wide, dorsal scutum very small. ***Epigastric area***: surface without external features (Fig. 15H). ***Endogyne***: from the middle of the slightly thickened margin of the postgastric scutum runs a dark, simple winding tube posteriorly, ending in a circular atrium (Fig. 23I, J).

#### Distribution.

China, Myanmar.

### Ischnothyreus sijiae

Taxon classificationAnimaliaAraneaeOonopidae

Tong & Li sp. nov.

C0B74775-280B-5832-B734-8976CB33CD60

http://zoobank.org/CD66D79E-1F25-4A40-8BF7-F1172AA82CDE

Figures 16 , 24A, B

#### Type material.

***Holotype*** ♀: China, Yunnan, Xishuangbanna Tropical Garden; 21°54.999'N, 101°16.237'E; 561 m; 24.IV.2019; Y. Tong and J. Liu leg. (SYNU-426). ***Paratype*.** 1♀: same data as for holotype (SYNU-427).

#### Diagnosis.

The new species is similar to *I. tadfane* Tong & Li, 2013 in the large dorsal scutum of the abdomen, but can be distinguished by the greater short winding tube of the endogyne (Fig. 24B) (vs long winding tube; Tong and Li 2013: fig. 4I, K) and the nipple-shaped atrium (Fig. 24A) (vs inverted bell shaped atrium; Tong and Li 2013: fig. 4H, J).

**Figure 16.**
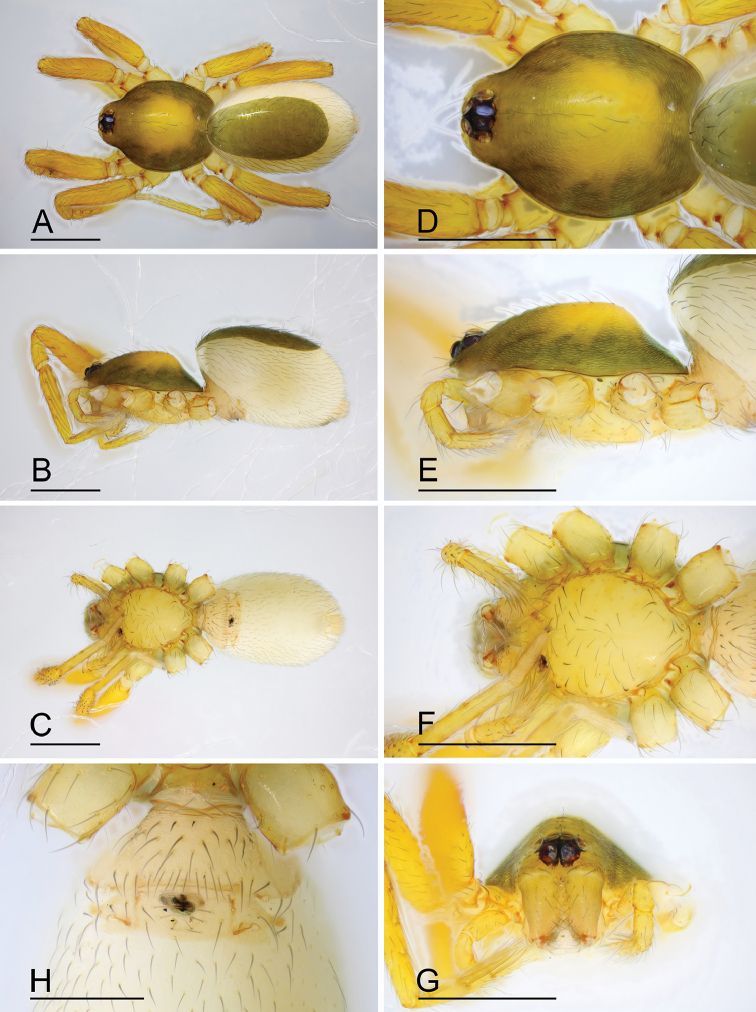
*Ischnothyreus sijiae* sp. nov., female paratype **A–C** habitus, dorsal, lateral and ventral views **D–G** prosoma, dorsal, lateral, ventral and anterior views **H** epigastric region, ventral view. Scale bars: 0.4 mm (**A–G**); 0.2 mm (**H**).

#### Description.

**Female (holotype). *Body***: habitus as in Fig. 16A–C; body length 1.49. ***Carapace***: 0.68 long, 0.54 wide; yellow-brown, without any pattern, surface finely reticulate, lateral margin straight, smooth (Fig. 16D, E). ***Clypeus***: height about 0.8 times ALE diameter (Fig. 16G). ***Eyes***: see Fig. 16D, G. ***Sternum***: pale orange (Fig. 16F). ***Mouthparts***: chelicerae, endites and labium orange; chelicerae straight, base of fangs unmodified (Fig. 16G); endites unmodified (Fig. 16F). ***Abdomen***: 0.86 long, 0.53 wide; dorsal scutum well sclerotized, brown, covering 2/3 of the abdomen width and approximately 4/5 of the abdomen length, not fused to epigastric scutum; epigastric and postgastric scutum well sclerotized, pale orange, unfused (Fig. 16A–C). ***Legs***: pale orange, femur I with 2 prolateral spines, tibia I with 4 pairs, metatarsus I with 2 pairs of long ventral spines. Leg II spination similar to leg I except femur with only 1 prolateral spine. Legs III and IV spineless. ***Epigastric area***: surface without external features (Fig. 16H). ***Endogyne***: from the middle of the slightly thickened margin of the postgastric scutum runs a dark, simple winding tube, ending in a small nipple-shaped atrium (Fig. 24A, B).

**Male.** Unknown.

#### Etymology.

The species is named after Miss Sijia Liu, one of the collectors of the holotype.

#### Comment.

Among the known species of the genus *Ischnothyreus* of China, *I. qianlongae* Tong & Li, 2008 and *I. tergemintus* Liu, Xu & Henrard, 2019 are known only from males. *I. sijiae* sp. nov. can be distinguished from *I. tergemintus* by the brown colour on the abdomen and sides of carapace (vs yellow abdomen and carapace in *I. tergemintus*; Liu et al. 2019: fig. 1A); and from *I. qianlongae* by the smaller size of the eyes (compare Fig. 16D with Tong and Li (2008: fig. 4A)).

#### Distribution.

Known only from the type locality.

### Ischnothyreus xiaolongha

Taxon classificationAnimaliaAraneaeOonopidae

Tong & Li sp. nov.

DC2D9515-3CFD-5E58-89FD-016FEA7539A5

http://zoobank.org/052DD21A-D656-4E9E-84B5-6963D3D65BA6

**Figure 17.**
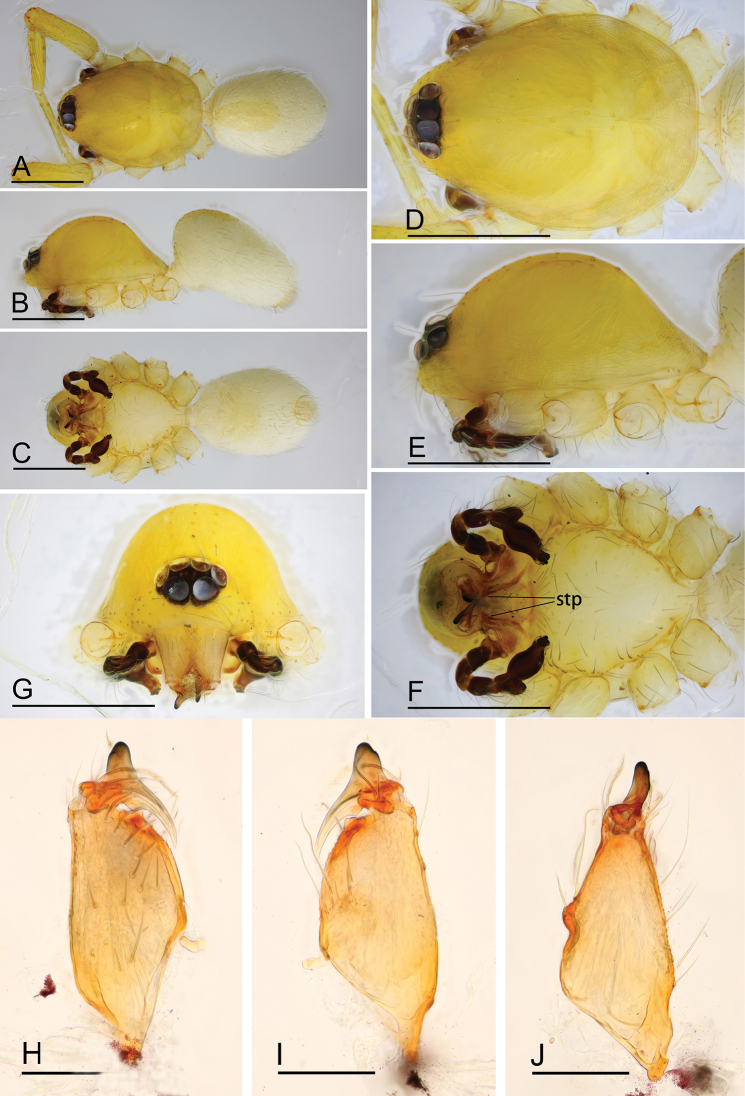
*Ischnothyreus xiaolongha* sp. nov., male holotype **A–C** habitus, dorsal, lateral and ventral views **D–G** prosoma, dorsal, lateral, ventral and anterior views **H–J** left chelicerae, anterior, posterior and lateral views. Abbreviation: stp = strong, tooth-like projection. Scale bars: 0.4 mm (**A–G**); 0.1 mm (**H–J**).

**Figure 18.**
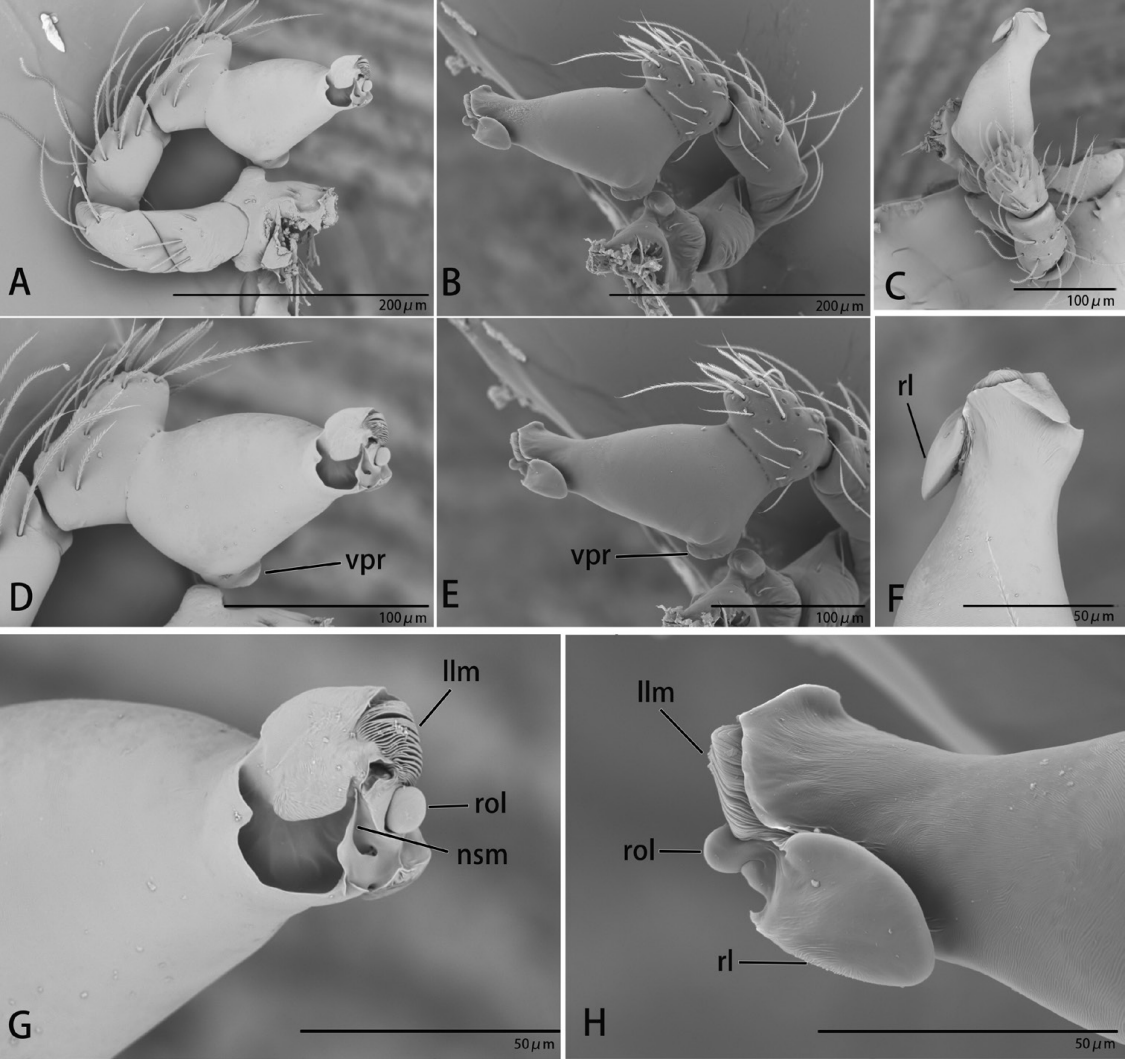
*Ischnothyreus xiaolongha* sp. nov., male holotype, left palp, SEM**A–C** prolateral, retrolateral and dorsal views **D, E** palpal bulb, prolateral and retrolateral views **F–H** distal part of palpal bulb, dorsal, prolateral and retrolateral views. Abbreviations: llm = lamella-like membrane; nsm = needle-shaped membrane; rl = retrolateral lobe; rol = round lobe; vpr = ventral protrusion.

**Figure 19.**
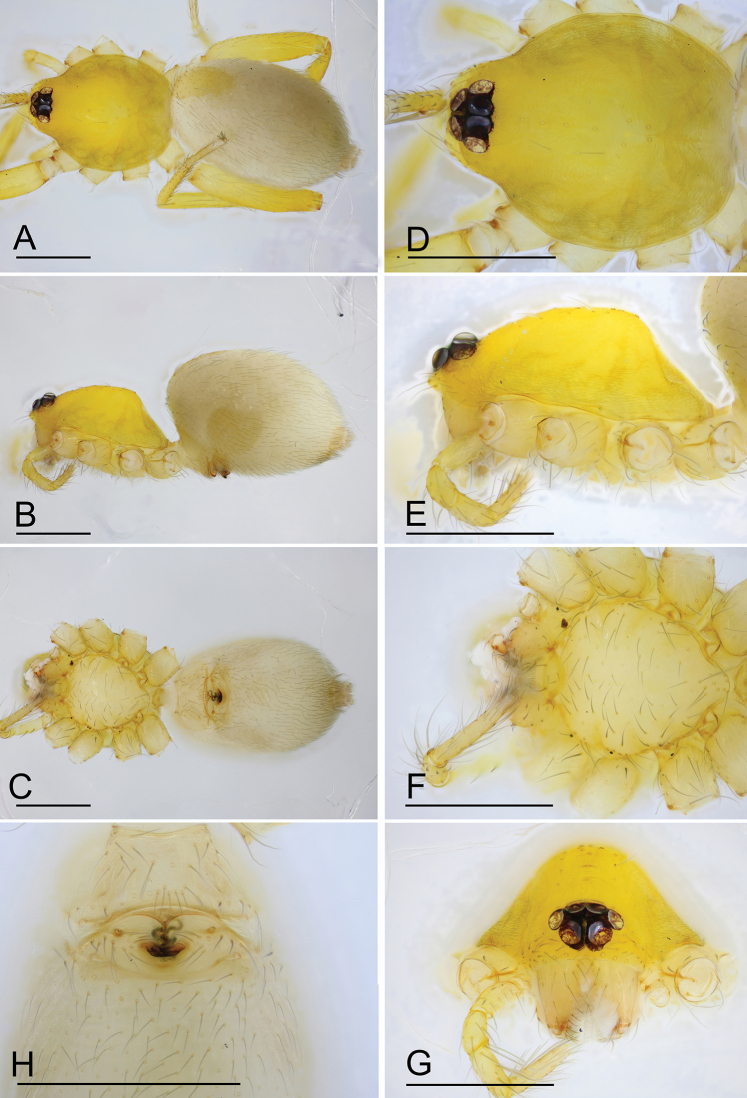
*Ischnothyreus xiaolongha* sp. nov., female paratype **A–C** habitus, dorsal, lateral and ventral views **D–G** prosoma, dorsal, lateral, ventral and anterior views **H** epigastric region, ventral view. Scale bars: 0.4 mm (**A–H**).

Figures 17 , 18 , 19 , 21A–E , 24C, D

#### Type material.

***Holotype*** ♂: China, Yunnan, Mengla County, Xiaolongha, Xishuangbanna Biodiversity Conservation Corridor, Qidaoban, valley forest; 21°24.832'N, 101°37.906'E; 721 m; 18.VI.2013; Q. Zhao and Z. Chen leg. (SYNU-428). ***Paratypes*** 1♀: same data as for holotype (SYNU-429); 1♂3♀: same data as for holotype; 21°24.253'N, 101°36.324'E; 761 m; 15.VI.2013; Q. Zhao and Z. Chen leg. (SYNU-430–433).

#### Diagnosis.

The new species is similar to *I. cristiformis* sp. nov. and *I. mangun* sp. nov. in the lamella-like membrane of the male palp and the short dorsal scutum of the abdomen, but can be distinguished by the tongue-shaped sclerotized process (Fig. 17H–J) of the cheliceral fang (vs large cockscomb-shaped sclerotized process (Fig. 1H–J) in *I. cristiformis* sp. nov. and the unmodified male cheliceral fang (Fig. 4H, I) in *I. mangun* sp. nov.), the broad leaf-shaped retrolateral lobe of the male palp (Fig. 18F) (vs broad rectangular-shaped (Fig. 2F) in *I. cristiformis* sp. nov. and narrow leaf-shaped (Fig. 5F) in *I. mangun* sp. nov.) and the small bell-shaped atrium (Fig. 19H) of the female (vs the large bowl-shaped atrium (Fig. 3H) in *I. cristiformis* sp. nov. and the small rectangular shaped atrium (Fig. 6F) in *I. mangun* sp. nov.).

#### Description.

**Male (holotype). *Body***: habitus as in Fig. 17A–C; body length 1.48. ***Carapace***: 0.80 long, 0.61 wide; yellow, with egg-shaped patches behind eyes, surface of elevated portion of pars cephalica smooth, sides finely reticulate, fovea absent, lateral margin straight, smooth (Fig. 17D, E). ***Clypeus***: height about equal to ALE diameter (Fig. 17G). ***Eyes***: see Fig. 17D, G. ***Sternum***: pale orange (Fig. 17F). ***Mouthparts***: chelicerae, endites and labium orange; chelicerae straight, base of fangs with tongue-shaped sclerotized process, fang groove with a few small denticles (Figs 17H–J, 21D, E); anteromedian tip of endites with one strong, tooth-like projection (Fig. 17F). ***Abdomen***: 0.64 long, 0.48 wide; dorsal scutum pale orange, very small, covering approximately 1/4 of the abdomen width and 1/3 of the abdomen length, not fused to epigastric scutum; epigastric and postgastric scutum well sclerotized, pale orange, fused, postgastric scutum covering about 1/2 of the abdomen length (Fig. 17A–C). ***Legs***: pale orange, femur I with 2 prolateral spines, tibia I with 4 pairs, metatarsus I with 2 pairs of long ventral spines. Leg II spination similar to leg I except femur with only 1 prolateral spine. Legs III and IV spineless. ***Palp***: trochanter with ventral projection, cymbium brown; bulb with 2 ventral protuberances, one large and another very small, distal end of bulb stout, with one narrow needle-shaped prolateral projection and a lamella-like membrane, retrolateral lobe broad leaf-shaped (Figs 18, 21A–C).

**Female (paratype, SYNU-429).** Same as male except as noted. ***Body***: habitus as in Fig. 19A–C; body length 1.70. ***Carapace***: 0.76 long, 0.63 wide. ***Mouthparts***: chelicerae and endites unmodified. ***Abdomen***: 0.95 long, 0.64 wide. ***Epigastric area***: surface without external features (Fig. 19H). ***Endogyne***: from the middle of the slightly thickened margin of the postgastric scutum runs a dark, simple winding tube posteriorly, ending in a small bell-shaped atrium (Fig. 24C, D).

#### Etymology.

The specific name is a noun in apposition taken from the type locality.

#### Distribution.

Known only from the type locality.

**Figure 20.**
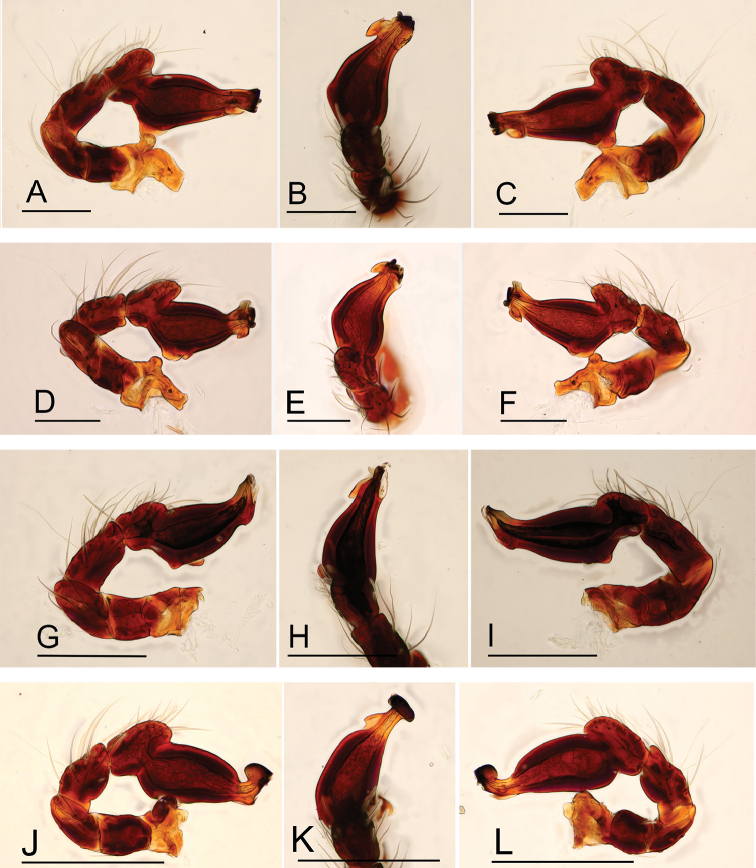
*Ischnothyreus* spp., left male palp **A–C***I. cristiformis* sp. nov. **D–F***I. mangun* sp. nov. **G–I***I. mengyang* sp. nov. **J–L***I. qidaoban* sp. nov. **M–O***I. qiuxing***A, D, G, J, M** prolateral view **B, E, H, K, N** dorsal view **C, F, I, L, O** retrolateral view. Scale bars: 0.1 mm (**A–F, M–O**); 0.2 mm (**G–L**).

**Figure 21.**
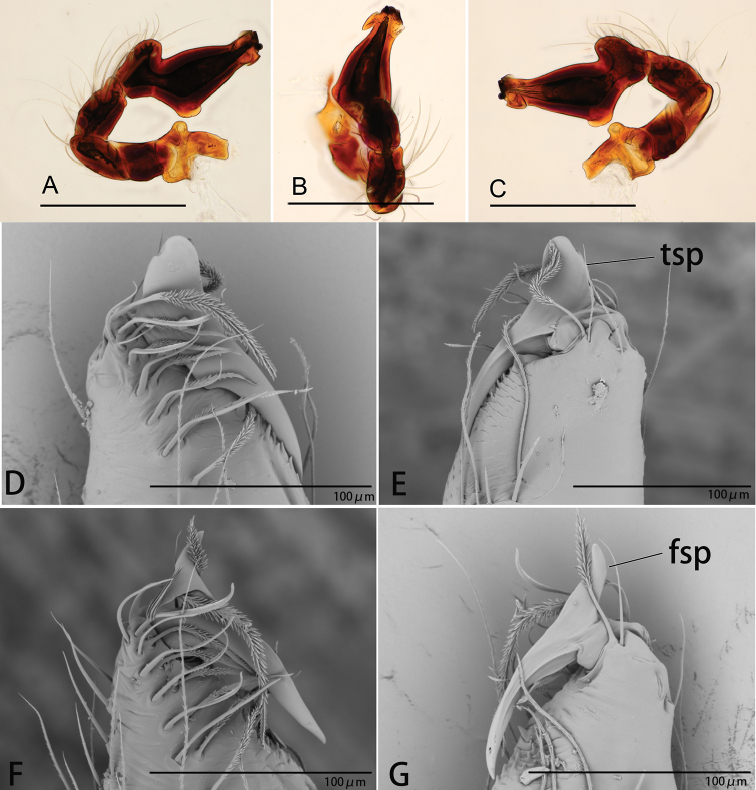
**A–E***I. xiaolongha* sp. nov. **F, G***I. qiuxing***A** left male palp, prolateral view **B** left male palp, dorsal view **C** left male palp, retrolateral view **D, F** left male chelicerae, anterior view **E, G** left male chelicerae, posterior view. Abbreviations: fsp = finger-shaped process; tsp = tongue-shaped process. Scale bars: 0.2 mm (**A–C**).

**Figure 22.**
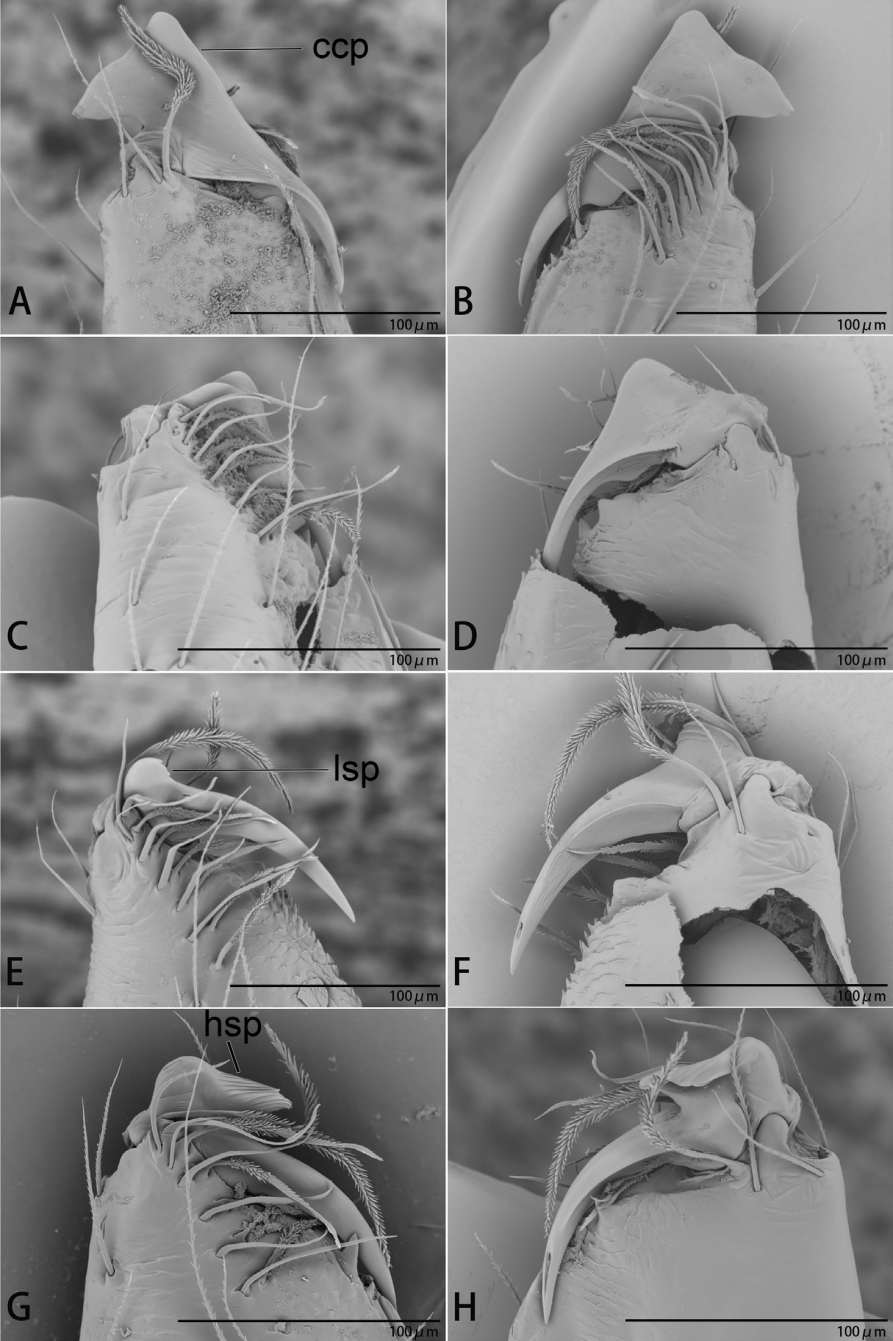
*Ischnothyreus* spp., left male chelicerae **A, B***I. cristiformis* sp. nov. **C, D***I. mangun* sp. nov. **E, F***I. mengyang* sp. nov. **G, H***I. qidaoban* sp. nov. **A, C, E, G** anterior view **B, D, F, H** posterior view. Abbreviations: ccp = cockscomb-shaped process; hsp = helmet-shaped process; lsp = large, sclerotized process.

**Figure 23.**
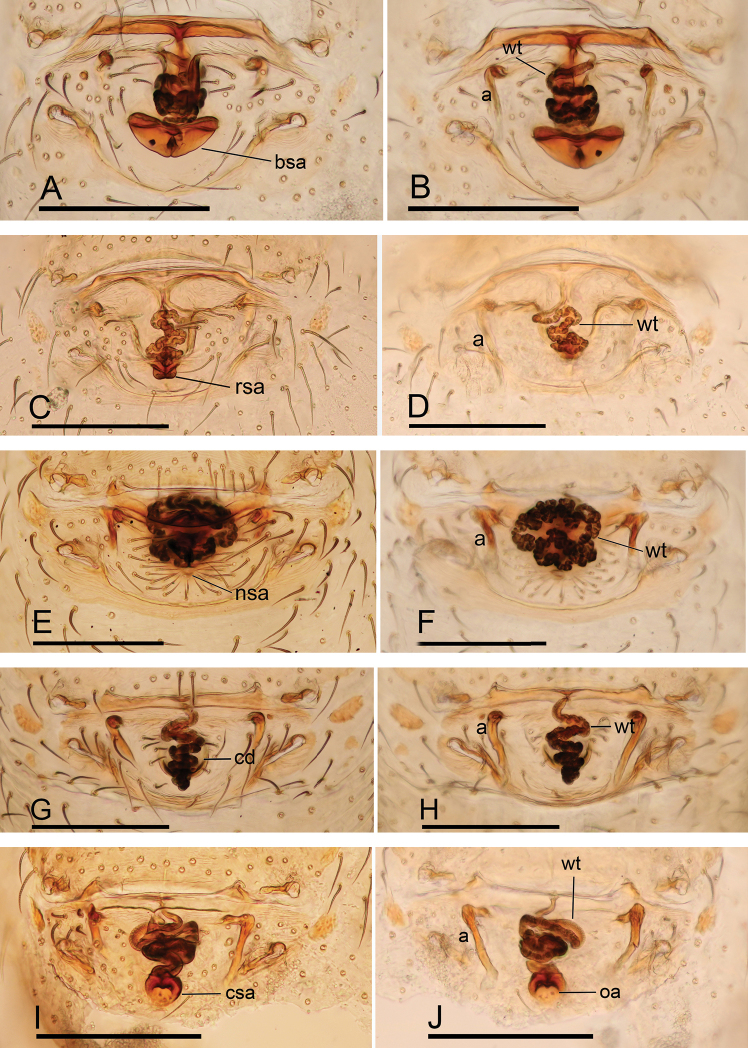
Endogyne of *Ischnothyreus* spp., **A, B***I. cristiformis* sp. nov. **C, D***I. mangun* sp. nov. **E, F***I. mengyang* sp. nov. **G, H***I. qidaoban* sp. nov. **I, J***I. qiuxing***A, C, E, G, I** ventral view **B, D, F, H, J** dorsal view. Abbreviations: a = apodemes; bsa = bell-shaped atrium; cd = central oval depression; csa = circular atrium; nsa = nipple-shaped atrium; oa = opening of the atrium; rsa = rectangular-shaped atrium; wt = winding tube. Scale bars: 0.2 mm.

**Figure 24.**
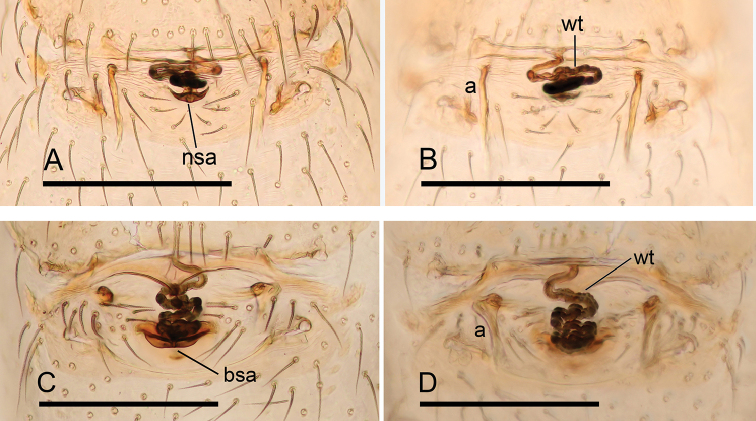
Endogyne of *Ischnothyreus* spp., **A, B***I. sijiae* sp. nov. **C, D***I. xiaolongha* sp. nov. **A, C** ventral view **B, D** dorsal view. Abbreviations: a = apodemes; bsa = bell-shaped atrium; nsa = nipple-shaped atrium; wt = winding tube. Scale bars: 0.2 mm.

## Supplementary Material

XML Treatment for Ischnothyreus cristiformis

XML Treatment for Ischnothyreus mangun

XML Treatment for Ischnothyreus mengyang

XML Treatment for Ischnothyreus peltifer

XML Treatment for Ischnothyreus qidaoban

XML Treatment for Ischnothyreus qiuxing

XML Treatment for Ischnothyreus sijiae

XML Treatment for Ischnothyreus xiaolongha
